# Longer operative times but equally safe - a propensity score-matched analysis for laparoscopic procedures in women with early-stage (r-ASRM I-II) endometriosis performed by gynecology residents compared to attending surgeons

**DOI:** 10.1186/s12909-025-06952-y

**Published:** 2025-03-27

**Authors:** Mona W. Schmidt, Anja Rosin, Sabine Schmidt, Joscha Steetskamp, Valerie C. Linz, Karin Rodewald, Lina Schiestl, Katharina Gillen, Marco J. Battista, Kathrin Stewen, Annette Hasenburg, Roxana Schwab

**Affiliations:** Department of Gynaecology and Obstetrics, University Medical Centre Mainz, Langenbeckstraße 1, 55131 Mainz, Germany

**Keywords:** Endometriosis, Safety, Residents, Operative time, Surgical experience, Costs of surgery, Medical education

## Abstract

**Introduction:**

Surgery for endometriosis is usually performed through minimally invasive surgery, either by experienced endometriosis surgeons or by supervised gynecology residents during their surgical training. This trial aimed to assess the influence of surgical experience on the efficiency and safety of minimally invasive surgery treatment for early-stage endometriosis.

**Material and Methods:**

Post- and introperative complications rates and length of stay of patients with stage I and II (revised American Society of Reproductive Medicine stage (rASRM)) endometriosis undergoing laparoscopic surgery at the University Hospital Mainz, Germany, between 2018 and 2022 were evaluated in a propensity score-matched analysis based on the experience of the primary surgeon (resident/fellow vs. attending). Linear and logistic regression models were used on the matched data set to calculate the treatment effect on the treated.

**Results:**

580 patients were included in the final data set. Of those, 339 were operated on by 11 attending surgeons and 241 by 22 residents/fellows. The matched dataset showed a mean difference of 0.02 in propensity scores after full propensity score-matching. Compared to surgical procedures performed by experienced surgeons, prolonged operating times were found for surgeries performed by residents/fellows (5.27 min in the whole data set (SE 1.36), *p* < 0.001), and 9.54 min (SE 3.57, *p* = 0.007) when analyzing only rASRM stage II endometriosis. The need for revision surgery was reduced in the resident/fellow group, but did not reach statistical significance (0.56 (95%CI: 0.301-0.1.02), *p* = 0.06). No significant differences were found for intra- or postoperative complications and length of hospital stay.

**Conclusions:**

Gynecology residents and fellows trained on the patient can safely perform surgery for early-stage endometriosis at the cost of increased operative times. Additional training options, such as surgical simulation training, should be explored to shorten learning curves, reduce the financial burden on hospitals due to prolonged operative times and counter the impending reduction in intraoperative training possibilities for residents.

## Background

Endometriosis is one of the most common benign gynecologic diseases. With an estimated prevalence of 10–15% in women of reproductive age, the psychological and physiological strain of this disease should not be underrated [1]. Common symptoms of endometriosis include chronic pelvic pain, dyspareunia, dysmenorrhea and hypermenorrhea. Furthermore, many women suffer from infertility [2]. Due to the unspecific symptoms, the diagnosis is often delayed by more than 9.6 years in Germany [1, 3]. Diagnostic and therapeutic options often rely on minimally invasive surgery (MIS), with many patients undergoing laparoscopic removal of extragenital endometrial tissue multiple times during their fertile life.

Despite many technological advances in surgical simulation training, most residents still gather their surgical expertise directly in the operating room under the supervision of a more experienced surgeon. This mentor-mentee ship approach has dominated the surgical field since the beginning. Even in the late 19th century an approach of “trial and error” on patients was not uncommon. As a pioneer in surgical education, William Halsted introduced the first structured training curriculum, following his principal “see one, do one, teach one” [4]. This concept relies on a stepwise increase in surgical independence of the trainee and one can argue that this approach is still the most commonly used teaching method in surgery. However, with the advancement in surgical techniques, the development of new surgical approaches and simulation options, one can argue that Halsted’s maxim might not be up-to-date and ethical anymore [5]. Our patient´s safety should be of the highest priority and with laparoscopy, the instruction through experts is often limited to verbal instructions, thus decreasing the control of the experienced surgeon over the learning surgeons’ actions in the operating room. However, MIS simultaneously increases the potential for surgical education. Through the magnification of the operative screen with the use of a camera, the attending surgeon can show the learning surgeon where to cut or how to position the instrument very accurately. This benefit especially comes to light in training centers, where in an unsterile field the attending surgeon can point directly on the screen. In 2013, Birkmeyer et al. demonstrated the influence of surgical skill on complication and mortality rates after bariatric bypass surgery. Complication rates were almost three times as high in the less skilled group compared to highly skilled surgeons [6]. Being less complicated, early-stage endometriosis is often considered a straightforward surgical procedure for gynecology residents training to learn MIS. Thus, the question arises: how safe is it for patients with early-stage endometriosis to have their procedure performed by gynecology residents who learn directly on the patient?

To answer these questions, this trial aims to compare the operative time, the intra-/perioperative complications and the length of stay at the hospital in a propensity score-matched population between residents and attending physicians.

## Materials and methods

### Study setting and patient population

Women undergoing laparoscopic surgery with resection of extragenital endometrial tissue between 2018 and 2022 at the University Medical Center of the Johannes Gutenberg-University Mainz were eligible for this study. Patient data, including baseline characteristics, surgical parameters and postoperative complications were extracted into a preset spreadsheet. Surgical reports, anesthesiology reports, histopathological reports, and medical charts were searched to retrieve the necessary data. The #ENZIAN classification [7] was introduced in 2021 (additionally including a scoring system for superficial endometriosis); for all surgeries before 2021, the #ENZIAN was determined by the original ENZIAN classification (only deep-infiltrating endometriosis) [8], as well as by considering surgical and histopathological reports. The #ENZIAN classification includes superficial lesions at the peritoneum (P), ovaries (O), assessed the tubo-ovarian condition (T), as well as deep-infiltrating endometriosis lesions in the rectovaginal space/vagina/retrocervical area (A), sacrouterine ligaments/cardinal ligaments/pelvic sidewall (B), rectum (C) and other sites (F). As it was not possible to reliably determine the tubo-ovarian condition due to varying surgical report descriptions, it was disregarded from this analysis.

To achieve a more homogenous data set and thus, to allow for comparability of the patient populations between both groups and limit the influence of major confounders in our dataset, the final selection of cases was based on the following inclusion criteria:

Inclusion:

- Endometriosis stage I or II (according to the revised American Society of Reproductive Medicine (rASRM) stage) [9].
- None or only the following small simultaneous operative procedures: diagnostic hysteroscopy, insertion or extraction of an intrauterine pessar, salpingectomy, adnexectomy, removal of pedunculated uterine fibroids.
- Diagnostic laparoscopies, where endometriosis was diagnosed but not removed.

Exclusion:

- Simultaneous other surgical procedures such as hysterectomy, operative hysteroscopy, enucleation of myomas, abdominal wall resection of endometrial tissue.
- (Pre) cancerous lesions.
- Extensive surgeries including multidisciplinary and multivisceral surgical resections (e.g. urology or general surgery).

### Patient characteristics and outcomes

All data points extracted for analysis can be found in Table 1. The experience level of surgeons was divided into resident and fellow surgeons compared to laparoscopically experienced attending surgeons.

**Table 1:** Extracted data points

Category	Extracted data points
Patient characteristics	Age at date of surgery
	Body Mass Index
	Prior abdominal surgery score*
Stage of endometriosis	rASRM stage
	#ENZIAN classification
Surgical parameters	Experience of the surgeon
	Operative time
	Surgical procedures *(peritonectomy*,* adhesiolysis*,* ureterolysis*,* removal of ovarian cysts*,* salpingectomy*,* adnexectomy*,* chromopertubation*,* diagnostic hysteroscopies*,* other minor surgical procedures*)
	Intraoperative complications *(bowel injury*,* bladder injury*,* other injuries)*
Postoperative parameters	Postoperative complications
	Length of hospital stay

* Two points for each prior laparotomy or cesarean section, one point for each laparoscopy based on Hildebrandt et al. [10]

rASRM stage = revised American Society of Reproductive Medicine stage;

### Educational regime

Comparable to the vast majority of hospitals, gynecologic residents and fellows within this trial are introduced to laparoscopic surgery in a stepwise approach during their residency. Commonly, residents assisted in laparoscopic surgeries by guiding the camera and observing laparoscopic procedures starting in their first year of training. When needed residents are also allowed to guide another instrument to assist, most often by holding certain structures. In general, the educational curriculum in our hospital involves residents rotating through other areas, including obstetrics, gynecologic oncology and outpatient services during their 2nd to 4th year of training, severely limiting the time spent in the operating room. In the 5th year of training, they rotate back to the operating room for a more focused surgical education. During this year, residents are trained to assist more actively in laparoscopic procedures and are allowed to excise some endometriosis lesions in low-risk areas before performing their first series of surgical procedures as the primary surgeon, which are frequently early-stage endometriosis procedures. Throughout the residency, an attending surgeon or experienced fellow is always present to assist during all surgical procedures. As a young fellow, these simple procedures are often performed with the assistance of an experienced resident, but also with attending surgeons.

### Statistical analysis

For the primary analysis, the data set was split according to the experience level of the primary surgeon (residents + fellows vs. attending surgeons). Characteristics of included patients, surgical procedures, and complications were reported as mean (± standard deviation) or median (interquartile range) where appropriate.

To assess the influence of the surgeon’s experience on the length of operative times and to account for imbalances in the data set due to the increased difficulty of cases performed by experienced surgeons, a propensity score matched analysis was performed. In a first step, a linear regression analysis was performed to identify factors influencing operative times. Patients were then matched according to these significant, as well as non-significant but clinically relevant characteristics, using the MatchIt package in R. 1:1 nearest neighbor matching and full matching are explored to identify the best method to achieve balanced groups. The maximum distance (caliper width) for an acceptable match was set at 0.05. Propensity scores were estimated using logistic regression of the treatment on the covariates. To estimate the average treatment effect on the treated and its standard error, a linear regression model (for continuous outcomes) and a logistic regression model (for categorical outcomes) were used, including the weights for all covariates determined through the matching process.

Statistical analyses were performed using StataBE 17 V5, propensity score matching and calculation of treatment effects were performed in RStudio based on R version 4.3.1. An α of 0.05 was regarded as statistically significant.

## Results

### Study population

A total of 582 patients fulfilled the eligibility criteria. Of those 215 procedures were performed by gynecology residents, 26 by gynecologists, 20 attending gynecologists without a surgical focus on endometrioses and 321 by attending surgeons with a surgical focus on endometriosis. Overall, 352 had an rASRM stage I and 230 women presented with an rASRM stage II. Patients whose surgery was performed by attendings differed from those performed by residents or fellows clinically relevant with regards to the number of previously performed abdominal surgeries, the rASRM stage and the presence of deep endometriosis. Ureterolysis and chromopertubation were performed more by attending surgeons, whilst more diagnostic hysteroscopies and removal of ovarian cysts were performed by residents and fellows (see Table 2).

**Table 2:** Population characteristics of the original data set

	Residents and fellows *N* = 241	Attending physicians *N* = 341
**Age**	29.50 ± 6.70	29.87 ± 7.02
**Operative time**	56.44 ± 24.31	59.76 ± 27.28
**BMI**	24.50 ± 5.03	23.85 ± 5.06
Previous abdominal procedure score* 0 1 2 3 4 5 > 5	150 (62.2%) 56 (23.2%) 19 (7.9%) 12 (5.0%) 2 (0.8%) 1 (0.4%) 1 (0.4%)	189 (55.8%) 88 (26.0%) 33 (9.7%) 9 (2.7%) 8 (2.4%) 9 (2.7%) 3 (0.9%)
**ASA score** 1 2 3 4	61 (25.7%) 172 (72.6%) 3 (1.3%) 1 (9.4%)	92 (27.1%) 238 (70.2%) 9 (2.7%) 0 (0%)
**rASRM stage** I II	170 (70.8%) 70 (29.2%)	182 (53.4%) 159 (46.6%)
**Deep endometriosis**	104 (43.2%)	204 (59.8%)
**Length of hospital stay**	2.95 ± 0.78	3.08 ± 0.74
**Operative steps**		
Chromopertubation	162 (67.5%)	254 (74.7%)
Removal of ovarian cyst	40 (16.6%)	43 (12.6%)
Salpingectomy	10 (4.1%)	18 (5.3%)
Ureterolysis	48 (19.9%)	116 (34.1%)
Diagnostic hysteroscopy	36 (14.9%)	25 (7.3%)
Adnexectomy	2 (0.8%)	2 (0.6%)
Cystoscopy/rectoscopy Bowl shaving	5 (2.1%) 3 (1.2%)	4 (1.2%) 7 (2.1%)
Other procedures	12 (5.0%)	19(5.6%)

* Two points for each prior laparotomy or cesarean section, one point for each laparoscopy based on Hildebrandt et al. [10]

ASA = American Society of Anesthesiologists physical status classification, rASRM stage = revised American Society of Reproductive Medicine stage

### Propensity score matching

After performing a multivariate linear regression model, the confounders removal of ovarian cyst, salpingectomy, uterolysis, hysteroscopy, other surgical procedures, rASRM stage, as well as the factors P, O, A, B, C and F of the #Enzian score remained as independent predictors of operative time and where included in the propensity score model. Due to missing data in two cases, 580 were included in the propensity score-matched analysis. All procedures were performed by a total of 22 gynecology residents/fellows and 11 gynecology attendings.

**Fig. 1:**
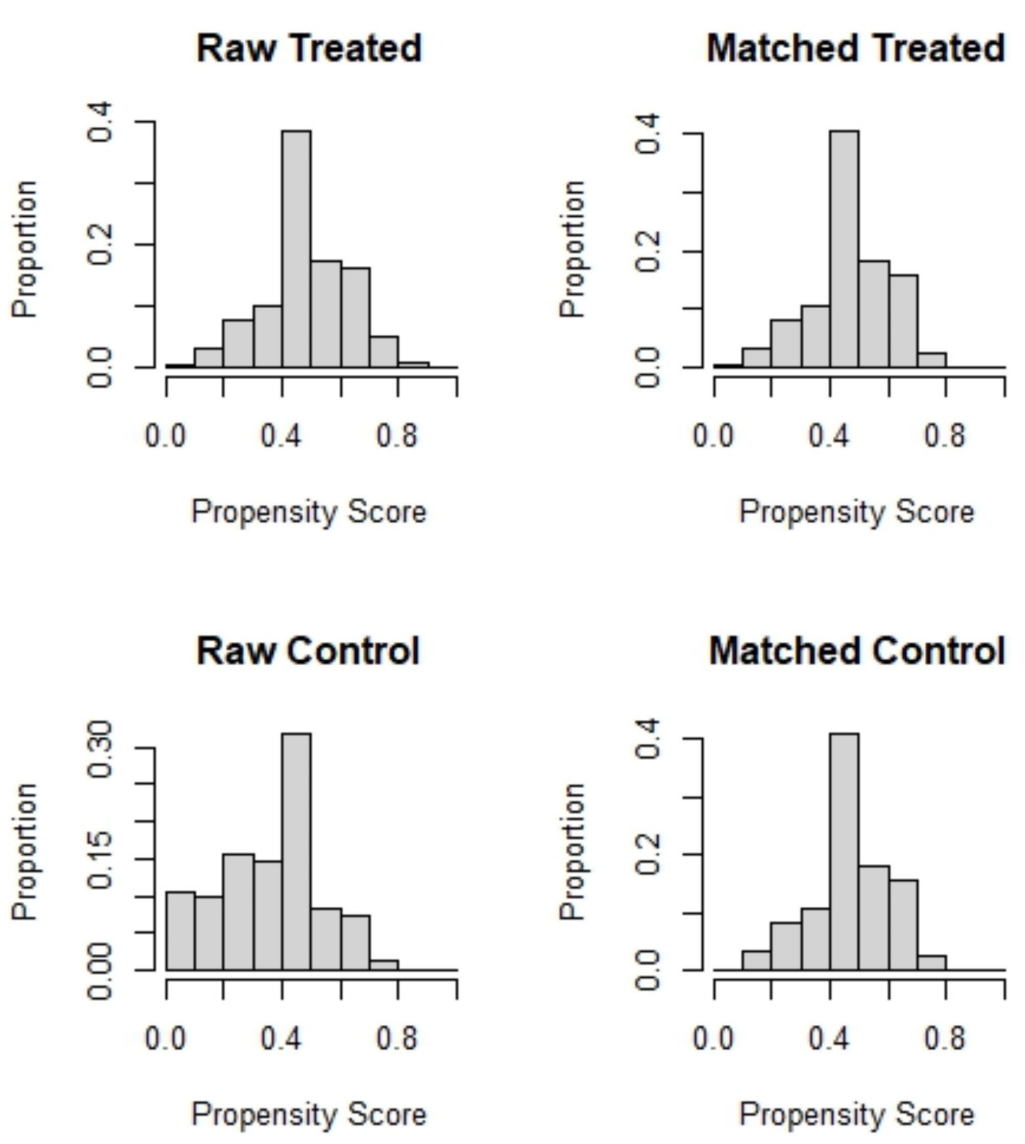
Propensity score balance before and after matching

**Fig. 2:**
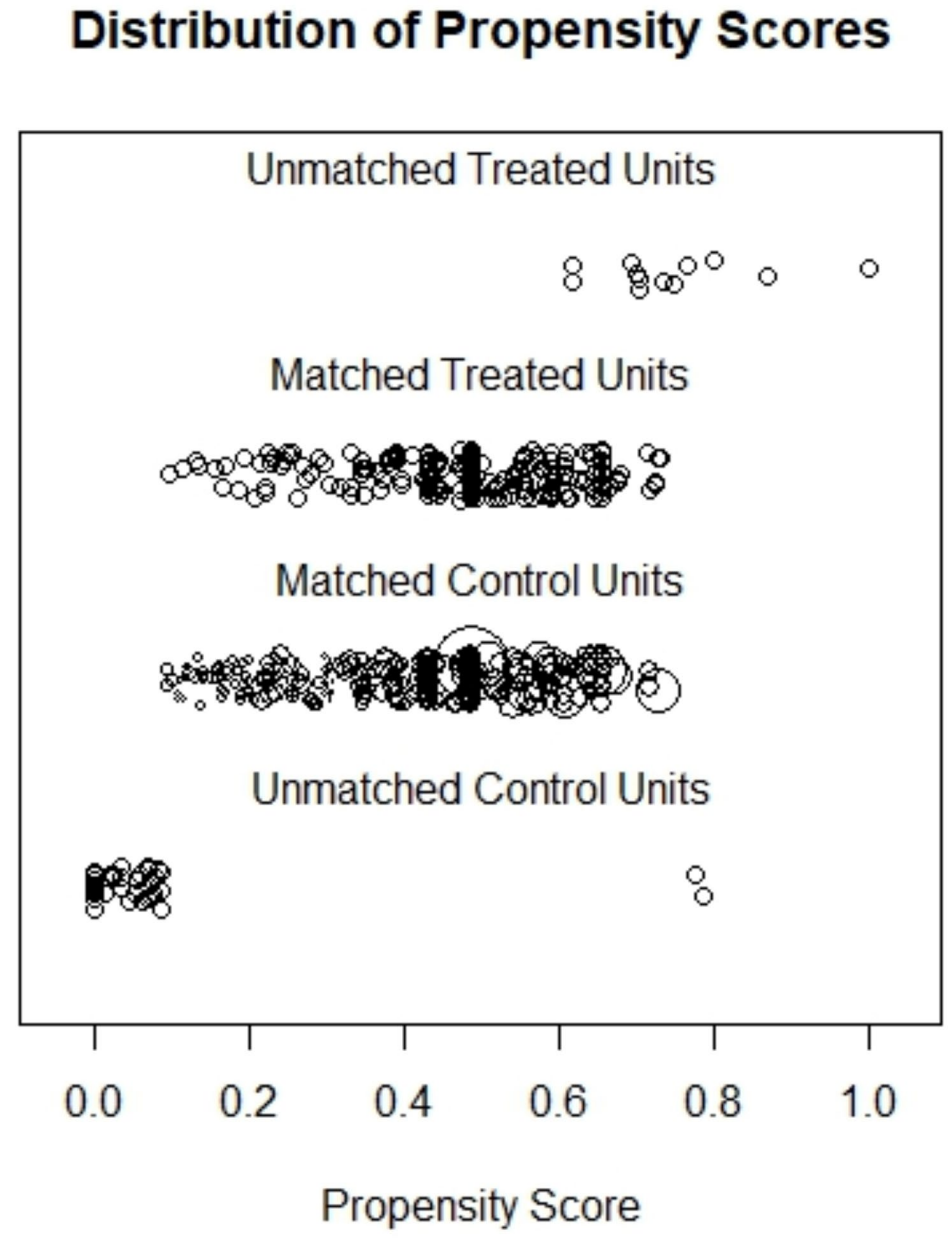
Distribution of Propensity Scores after matching

When checking baseline balances of all evaluated confounders, a standardized mean distance of 0.891 was found, indicating a substantial difference between both groups. After conducting a nearest neighbor-propensity score matching, a good improvement was seen with a remaining standardized mean difference of 0.007. However, around 192 cases were disregarded with this matching method. Thus, a full matching -propensity score matching method was performed in order to further reduce the standardized mean difference to 0.002 with 532 cases included in the matched data set (see Table 3). Due to the better balance and more comprehensive inclusion of the data set, the full matching-propensity score matching method was used for all further analyses. The increase in balance after the full match and the chosen cases can be seen in Figs. 1 and 2.

**Table 3:** Mean differences for confounding factors

	All data Attendings *n* = 339 Residents/Fellows *n* = 241	Nearest Neighbor Attendings *n* = 194 Residents/Fellows *n* = 194	Full matching Attendings *n* = 303 Residents/Fellows *n* = 229
**Mean distance**	0.891	0.007	0.002
**Removal of ovarian cysts** None Unilateral Bilateral	-0.113 0.117 -0.006	-0.042 0.042 0	-0.023 0.031 -0.030
**Salpingectomy** None Unilateral Bilateral	0.058 -0.154 0.034	0 0 0	-0.070 0.025 0.066
**Ureterolysis** None Unilateral Bilateral	0.351 -0.029 -0.650	-0.052 0.113 -0.103	0.130 0.178 -0.065
**Hysteroscopy** No Yes	-0.212 0.212	-0.029 0.029	0.012 -0.012
**Other procedures** None Very short Short	0.002 0.031 -0.071	0.024 -0.026 0	0.022 -0.021 -0.008
**rASRM stage** I II	0.380 -0.380	-0.011 0.011	0.058 -0.058
**#ENZIAN classification** P0 P1 P2 P3 O0 O1 O2 O3 O4 A0 A1 A2 A3 B0 B1 B2 B3 B4 B6 C0 C1 C2 C3 F0 F Adenomyosis F Bladder F Intestinum F Ureter F Other	0.061 0.256 -0.1972 -0.244 0.018 0.038 -0.095 -0.027 0.065 0.385 -0.125 -0.413 -0.176 0.341 -0.016 -0.342 -0.266 -0.260 -0.101 0.011 0.072 -0.247 -0.071 0.042 0.018 -0.020 -0.101 0.019 -0.104	-0.018 -0.011 0 0.044 -0.017 0 0.029 0 0 0.042 -0.080 0.052 0 -0.022 0.038 -0.019 -0.057 0.047 0 0 -0.022 0.080 0 0.069 -0.104 0 0 0 0.057	0.027 -0.026 -0.023 0.065 0.053 -0.059 -0.005 -0.024 0 0.062 -0.042 -0.045 0 0.025 -0.002 -0.034 -0.023 0.004 0 0.007 -0.015 0.028 0 0.217 -0.189 -0.042 0 -0.204 0.010

rASRM stage = revised American Society of Reproductive Medicine stage

#ENZIAN classification: superficial lesions at the peritoneum (P), ovaries (O), deep-infiltrating endometriosis lesions in the rectovaginal space/vagina/retrocervical area (A), sacrouterine ligaments/cardinal ligaments/pelvic sidewall (B), rectum (C) and other sites (F); the scores for both sides were added together for O and B, respectively

### Operative time

The operative time in surgeries performed by residents/fellows vs. attending physicians was similar in the unmatched group 56.4 ± 24.3 min (residents/fellows) vs. 59.8 ± 27.3 min (attendings), *p* = 0.131, respectively. In the matched cohort, the operative time was significantly longer for surgeries performed by residents or fellows (5.27 min (SE 1.36), *p* < 0.001), with a predicted operative time of 50.7 min (SE 1.50), compared to those performed by attending surgeons (56.0 min (SE1.47)). This represents a 10.5% increase in operative time in surgeries performed by residents and fellows compared to the operative time of experienced surgeons.

When analyzing only patients with rASRM stage II (*n* = 227) within the matching process (all other matching criteria remained the same), an increase of 9.54 min (SE 3.57, *p* = 0.007) in operative time was seen when residents/fellows performed the surgery (matched cohort: *n* = 58 residents/fellows, *n* = 116 attendings). This equals a 15.4% increase in operative time for surgical procedures performed by residents/fellows.

### Length of stay

Similar to the unmatched cohort, the length of stay in the hospital was not influenced by the surgeon’s experience: -0.054 days (SE 0.0598).

### Intra-operative complications

In all 580 cases, only one bowel injury occurred in a surgery performed by an attending surgeon, and one small bowel injury occurred in the resident or fellow group. The bowl injury was detected intraoperatively in the group of attending physicians, and a visceral surgeon was consulted to repair the lesion within the primary surgery. The patient experienced no further postoperative complications due to this bowel lesion. In the resident/fellow group, the small bowel lesion was detected postoperatively after the occurrence of a four-quadrant peritonitis. During the surgical revision with the consultation of a visceral surgeon, the lesion was repaired through excision of the injured bowel area and the creation of a small bowel anastomosis. Overall, no other severe intraoperative complications occurred in either group (e.g. ureter lesion, bladder lesion) and no conversion to laparotomy occurred within the study population.

### Post-operative complications and surgical revision

Of the 580 cases, 13 postoperative complications were noted. Of those, ten occurred in the group of patients operated on by attending surgeons (2.9%) and four in the resident/fellow group (1.6%). These postoperative complications included postoperative intraabdominal hemorrhage (*n* = 6), a large abdominal wall hematoma/bleeding from a trocar incision site (*n* = 3), intensive pain (*n* = 2), retroperitoneal hematoma (*n* = 1), reversible damage due to the positioning on the operative table (*n* = 1) and a small bowel perforation with severe peritonitis (*n* = 1). After matching, these differences did not remain apparent with a non-significant relative risk of 0.57 (95%CI0.269; 1.21, *p* = 0.142) for postoperative complications when operated on by a resident or a fellow compared to an attending.

Ten patients underwent a second surgery and one patient had an additional third surgical procedure. Of those, seven revisions occurred in the unmatched attending group (2.05%) and four in the resident/fellow group (1.67%). After matching, the same tendency to a lower risk for surgical revisions in the resident/fellow group was seen with a relative risk of 0.56 (95%CI: 0.301-0.1.02). However, this result did not reach significance (*p* = 0.06).

## Discussion

The impact of surgical experience on efficiency and complication rates in early-stage endometriosis is of great importance not only for patients but also for hospitals aiming to maximize the utilization of their operating room capacities and staff. The propensity score-matched analysis compared the operating time, intra- and postoperative complications and LOS between surgeries performed by gynecology residents or fellows versus gynecology attendings for rASRM stage I or II endometriosis. A 10–15% prolongation of operative time for surgical procedures performed by gynecology residents and fellows was found compared to attendings. Safety profiles and LOS were similar across both groups.

Surgical training is a key component in the medical education during gynecology residency. However, surgical education is always accompanied by a learning curve. In MIS, these learning curves tend to be prolonged to open surgical procedures [10, 11] due to the more challenging aspects of MIS including an indirect view of the operating field, as well as the loss of haptics and counterintuitively moving long instruments [12, 13]. Despite good simulation options nowadays, surgical training, with all its difficulties, is still mostly performed directly on the patient. With this trial, we provide real-world data on the safety and efficiency of this approach, which is still standard in the vast majority of hospitals worldwide. The step-wise approach and constant supervision at Mainz University Hospital demonstrated equal safety with regards to intra- and postoperative complications for patients with early-stage endometriosis when performed by residents or fellows when compared to surgeries only performed by attendings. In contrast, in this trial a tendency of reduced operative risk in resident/fellow-led surgical procedures was found. One explanation for this effect could be a more observant and careful approach by the supervising attending. When operating with a less experienced surgeon, each cut is carefully evaluated by the residents themselves and the respective attendings, who, if in doubt, will stop the resident early on. However, in an attending-led surgery the confidence in their skills might be higher, and thus riskier cuts may be undertaken or may be performed faster, leading to an increased risk for complications without a second set of experienced eyes to evaluate the situation. Furthermore, patients had more prior abdominal surgeries in the attending group, reflecting a higher risk for complications. Increased BMI is also often regarded as a risk factor for intra- and postoperative complications. Notably, in our data set, the mean BMI was slightly higher in the resident/fellow group. This highlights, how even though sometimes regarded as outdated, the mentor-mentee educational approach directly in on the patient still holds its value in today´s time. However, this does require uncomplicated, learning procedures to be available at every hospital, which, facing new structural changes in the German healthcare system might not be the case in every hospital anymore. In contrast to our results, Igwe et al. found an increased risk for reoperation rate and 30-day readmission rates with resident involvement in laparoscopic hysterectomies [14], while no difference in morbidity or mortality rates was found. While some studies report increased complication rates for surgical procedures due to resident involvement, most studies found resident involvement to be safe [15, 16].

With the constant pressure on hospitals to increase their revenue, the improvement of the utilization of operating capacities is of great importance. However, teaching surgical trainees is in direct contrast to this goal. Unfortunately, only scarce evidence exists on the impact of surgical experience on gynecologic procedures. In this propensity score-matched analysis, prolonged operative times by 10.5% in teaching procedures were found even in these allegedly easy and routine surgical procedures. For procedures in women presenting with higher stage endometriosis (rASRM II), this increased to 15.4% prolonged operative times in procedures performed by residents/fellows. These results are in line with recent data provided on laparoscopic hysterectomies. An analysis of 3441 patients who underwent laparoscopic hysterectomies was reported by Igwe et al. in 2014. They found a 32.3% increase in operating time through resident involvement in the surgery, compared to procedures performed by attendings alone [14]. In addition, Hildebrandt et al. found a 20.3% increase in operative time for laparoscopic hysterectomies performed by residents vs. attendings [17]. Interestingly, the increase in operating time was greater in a municipal maximum-care hospital (21.6 min) compared to a university hospital (10.2 min). The authors attributed this effect to different organizational processes in the respective institutions [17]. The financial costs for hospitals as a result of prolonged operative times differ vastly by the respective health care system and the number of staff involved. Thus, a specific calculation of costs is often complex. A research group in the US calculated an estimated financial cost of $53 million annually for the US healthcare system based on a 13-minute increase in operative time [18]. A recent study by Waeschle et al. found the costs of an OR minute (active operating time) for procedures lasting between 31 and 60 min to be around 16 Euros [19], including staff and anesthesia medication. This can vary by the number of surgeons involved in a case and was reported as an average for multiple surgical specialties, including gynecology. A reduction of 5.4 min/9.5 min means a reduction in costs of around 86 Euros/152 Euros per surgical procedure, respectively. While this does not seem like a considerable amount by itself, one has to keep in mind that this is a “beginner” surgical procedure and more complex procedures will lead to higher costs through the involvement of residents. To evaluate the accurate cost reductions/financial benefits through decreased operative times would need to incorporate a variety of factors e.g., the revenue of additional surgical procedures performed in the saved operative time, the direct patient care, the recruiting of new patients for surgical procedures and the reduction of overtime pay. However, this is beyond the scope of this work.

While the obvious impact of higher costs for hospitals is clear, the impact of these financial and time implications on surgical education for residents is worth discussing, especially in light of the currently often practiced “mentor-mentee” surgical education. Financial pressure on hospitals can lead to a reduction in staff e.g. the number of gynecologic residents. This in turn can lead to fewer opportunities to assist and observe surgical procedures. Especially in university hospitals, students in their last year of training or during their clinical clerkships can replace gynecologic residents in the OR, to free up the time of residents for ward labor or outpatient services, which cannot be covered through students. This significantly impacts the first step, “see one” of Halsteds’ approach. Furthermore, in recent years, the number of surgical procedures being performed as outpatient procedures has risen and with the upcoming reform of the German healthcare system will likely increase even more [20]. Thus, especially in university hospitals so-called “teaching” procedures will decrease, giving less opportunity to residents to perform their first surgical steps safely. Therefore, one might need to consider a different approach to educating gynecologic residents in the future to ensure the same safety observed within this trial. The European Association of Gynecological Endoscopy recommends that every hospital offering MIS to their patients needs a dry-lab to simulate surgical procedures and a structured training curriculum for all gynecology residents [21]. Surgical skills acquired in simulated training programs have been shown to transfer to the operating room, especially regarding surgical efficiency [22, 23]. So what to do now? Not involving residents in surgical procedures is not an option, as gynecology residents need to be trained to become surgically skilled attendings. Thus, at the University Hospital Mainz we have extended the surgical training as described in this trial by a training program in the newly implemented skills lab focusing on minimally invasive surgical skills. Whether or not, this will be able to substitute for the reduced OR time of gynecologic residents in the upcoming years or even improve the learning curve, safety and operative performance remains to be seen.

This study has limitations that must be considered when interpreting the results. First, this is a single-center retrospective data analysis. While the assessed parameters are primarily objective and cannot be changed (such as operative time, intraoperative complications, procedures performed and rASRM stage), the #ENZIAN score was only used in clinical routine from 05/2021. Prior to this date, the ENZIAN score was used, focusing on deep endometriosis, and disregarding peritoneal and ovarian lesions. Thus, for surgeries before this date, the #ENZIAN score was retrospectively defined based on the existing ENZIAN score, as well as the surgical and pathological reports. Through the use of the #ENZIAN score in the matching process, the presence of deep endometriosis lesions was taken into account. However, specific treatments of those lesions were not considered separately (such as whether a discoid or segmental resection in rectosigmoid endometriosis was performed) and could provide a bias in the trial results. Furthermore, postoperative complications were assessed based on the medical records of the university hospital where the procedure was performed. However, in the German health-care system, an outpatient gynecologist often performs routine follow-up care. Thus, this trial does not adequately report minor surgical complications, such as wound infection or prolonged slight pain. Especially long-term sexual, intestinal and urinary dysfunction, which can greatly impact the quality of life after deep endometriosis surgery [24] was not reliably reported and thus, can not be compared in this trial. Therefore, the influence of surgical skills on these outcomes cannot be answered within this trial. Nevertheless, it can be assumed that patients with major complications, such as postoperative hemorrhage, presented at our hospital or were sent by their outpatient gynecologist for a follow-up with their surgeon. While it can be expected, that the difference in surgical time taken and possibly also complications would increase in higher-stage, complex endometriosis patients (rASRM stage III-IV), these are not commonly performed predominantly by residents. Due to the small sample size of high-stage endometriosis cases with a resident as a lead surgeon, this paper is focused only on early-stage endometriosis cases (rASRM stage I-II) to decrease the risk of statistical bias.

In conclusion, surgery in women with early-stage endometriosis can be safely performed by gynecology residents and fellows at the cost of increased operative times of up to 5–10 min on average. This is an important message to patients worrying about the involvement of residents during their surgical treatment. Overall, this study highlights the safety of the current surgical training regime, using a step-wise approach under expert guidance. However, current and upcoming changes in the German health-care system can impact the quality and availability of surgical expert mentoring posing new challenges to surgical education to retain patients’ safety. Establishing surgical simulation centers and training curricula could offer a feasible and safe alternative and possibly even financially beneficial approach to further ensure a high-quality surgical education.

## Data Availability

The datasets generated and analysed during the current study are not publicly available due potential identifying factors compromising the privacy of patients, but are available from the corresponding author on reasonable request.

## Abbreviations

ASA score: American Society of Anesthesiologists physical status classification the ASA score is a subjective assessment of patient’s overall health that is based on five classes
A: Deep-infiltrating endometriosis lesions in the rectovaginal space/vagina/rectovaginal area (#ENZIAN classification)
B: Sacrouterine ligaments/cardinal ligaments/pelvic sidewall (#ENZIAN classification)
BMI: Body Mass Index
C: Rectum (#ENZIAN classification)
CI: Confidence interval
ENZIAN: The ENZIAN score, like the rASRM classification, is determined by the extent of endometriosis during surgery. It contains A, B, C, F, O, P and T
F: Other sites (#ENZIAN classification)
LOS: Length of (hospital) stay
MIS: Minimally invasive surgery
O: Ovaries (#ENZIAN classification)
OR: Odds Ratio
P: superficial lesions at the peritoneum (#ENZIAN classification)
rASRM: revised American Society of Reproductive Medicine stage
SE: Standard Error
StataBE: Stata Basic edition; For mid-sized datasets
T: Tubo-ovarian condition (#ENZIAN classification)
